# Competitive level differences in basic motor skills of wheelchair basketball players: a cross- sectional observational study

**DOI:** 10.3389/fphys.2026.1846446

**Published:** 2026-07-03

**Authors:** Miljan Hadžović, Stefan Stojanović, Tamara Ilić, Ana Lilić, Ismail Ilbak, Marko Aleksandrović, Zhencheng Li

**Affiliations:** 1 Faculty of Sport and Physical Education, University of Niš, Niš, Serbia; 2 Association of Wheelchair Basketball Clubs, Niš, Serbia; 3 Faculty of Sports Sciences, İnönü University, Üzümlü, Malatya, Türkiye; 4 Wheelchar Basketball Club “Nais”, Niš, Serbia; 5 College of Physical Education, Chongqing University, Chongqing, China

**Keywords:** agility, functional classification, functional trunk stability, speed, strength, wheelchair basketball players

## Abstract

**Introduction:**

Performance in wheelchair basketball largely depends on athletes’ physical performance, particularly basic motor abilities required for efficient wheelchair propulsion and movement during play. This study aimed to examine differences in basic motor abilities between wheelchair basketball players competing in Division B and Division C, both in the overall sample and within higher classification scores (3.0–4.5) and lower classification scores (1.0–2.5) groups.

**Methods:**

The sample included 52 wheelchair basketball players, members of the national teams of Serbia, Bulgaria, Montenegro, Croatia, and Bosnia and Herzegovina, aged 16–54 years, competing in Division B or C. Participants were grouped according to competitive level and functional classification. Basic motor abilities were assessed using nine field tests of speed, strength, and agility. Data were analyzed using descriptive statistics, the Kolmogorov–Smirnov test, *MANOVA* and one-way *ANOVA* (*p* < 0.05).

**Results:**

Multivariate analysis showed significant differences in basic motor abilities within Division B and Division C players overall and among players with lower classification scores. No significant multivariate differences were found between highly classified players. Univariate analyses indicated better speed and agility performance in Division B players, with no differences in strength. In the overall sample, Division B players performed significantly (p < 0.01) better than Division C players in speed tests (ST5m: 1.86 ± 0.18 vs. 2.02 ± 0.18 s; ST10m: 3.16 ± 0.29 vs. 3.39 ± 0.24 s; ST20m: 5.46 ± 0.38 vs. 5.82 ± 0.38 s) and agility tests (ATT: 14.50 ± 1.00 vs. 15.51 ± 1.35 s; DAT: 28.46 ± 1.48 vs. 30.98 ± 2.51 s; FET: 18.71 ± 1.12 vs. 17.08 ± 1.56). Among players with lower classification scores, Division B players also showed significantly better results in ST5m, ST20m, ATT, DAT, and FET (p < 0.05).

**Conclusion:**

The findings indicate clear differences in basic motor abilities between wheelchair basketball players of different competitive levels, particularly in speed and agility, with more pronounced differences among players with lower functional classification scores.

## 1 Introduction

Wheelchair basketball, as one of the most popular and attractive *parasports* for persons with disabilities (PWD) (Yüksel and Sevindi, 2018), has demonstrated a consistent increase in the number of professional players and sports organizations and attracts a growing audience worldwide (Cavedon et al., 2015). Success in wheelchair basketball largely depends on athletes’ physical performance (basic and sport-specific motor abilities), specifically on their ability to move efficiently, control the wheelchair, and respond to the physical demands of play (de Witte et al., 2016). Recent reviews also emphasize the importance of standardized field-based tests for assessing wheelchair basketball skills and physical fitness, particularly speed, passing, agility, and other wheelchair-specific abilities (Ferreira da Silva et al., 2022; Petrigna et al., 2022). In addition to the clearly defined *Official Wheelchair Basketball Rules* prescribed by the International Wheelchair Basketball Federation Executive Counsil (2024), adequate coach knowledge of each player’s characteristics and on-court performance is required (Mason et al., 2013). At the international level, wheelchair basketball is organized according to competition level and functional classification, with players classified from 1.0 to 4.5 points according to their functional capacity in the wheelchair; lower classification scores indicate greater functional limitation, whereas higher scores indicate greater trunk stability and mobility (International Wheelchair Basketball Federation Executive Counsil, 2024).

The specificity of wheelchair basketball as an intermittent *parasport* is reflected in the alternation of aerobic and anaerobic periods during play (Bloxham et al., 2001) and is characterized by a large number of sprints, frequent stopping of the wheelchair, slaloms, and changes of direction that require explosive strength (Vanlandewijck et al., 1999; Wang et al., 2005; Marszałek et al., 2019а). Recent studies based on training and competition load monitoring further indicate that wheelchair basketball performance should be interpreted in relation to game demands, external and internal load, and functional classification (Romarate et al., 2023; Hernández-Beltrán et al., 2024a, b). In this context, when structuring the training process, wheelchair basketball coaches must pay particular attention to exercises aimed at developing players’ speed, strength, and agility (Granados et al., 2015). It has been suggested that performance in wheelchair basketball largely depends on the ability to maneuver and control the wheelchair, i.e., agility (Frogley, 2010), and that player agility is influenced by the level of competition in which they participate (De Groot et al., 2012). Recent evidence also indicates that upper-limb strength is related to repeated change-of-direction ability in international-level wheelchair basketball players (Iturricastillo et al., 2022). Gil et al. (2015), indicate that experience in playing wheelchair basketball correlates with agility and technique, that time spent in civilian wheelchairs is associated with agility and speed, and that upper-limb force and strength correlate with player classification. More recent findings also show that initial push-rim propulsion outputs are associated with sprint performance and differ according to functional classification in elite wheelchair basketball players (García-Fresneda et al., 2022). Although differences in movement volume between playing positions in wheelchair basketball are known to be minimal (de Witte et al., 2016) and no significant differences have been reported in psychological characteristics between players from Division A and Division B, previous research indicates that competitive level may be associated with technical, tactical, and motor indicators, primarily endurance and coordination (Erdemir et al., 2009; Granados et al., 2015; Yüksel and Sevindi, 2018). In addition, physiological responses may differ between players with low and high classification scores (Marszałek et al., 2019a), which suggests that comparisons between competitive levels should also consider functional classification. Research on wheelchair basketball players lacks sufficient data on differences in basic motor abilities among elite players who are members of national teams and compete at international continental tournaments across different competitive levels (A, B, and C divisions of the European Championship); existing studies have largely focused on differences at the level of national championships and leagues.

Therefore, the aims of this study were to determine differences in basic motor abilities between wheelchair basketball players competing in Division B and Division C, as well as differences in relation to players’ functional trunk stability, that is, according to the classification-based division of players into those with high (from 3.0 to 4.5) and low (from 1.0 to 2.5) classification scores, but at different competitive levels. Such data may provide reference values for coaches and researchers, support more precise training planning and player evaluation, and help clarify how competitive level and functional classification are related to performance in elite wheelchair basketball. It is hypothesized that wheelchair basketball players in Division B will achieve better results than those in Division C in basic motor abilities, particularly speed, change-of-direction speed, and strength. It is also hypothesized that these differences between Division B and Division C players will be more prominent among players with lower classification scores (1.0–2.5) than among players with higher classification scores (3.0–4.5).

## 2 Methods

### 2.1 Participants

The sample in this study consisted of wheelchair basketball players who were members of the national teams of Serbia, Bulgaria, Montenegro, Croatia, and Bosnia and Herzegovina. Player (participant) classification was conducted based on the degree of disability and physical and functional mobility in the wheelchair, in accordance with the official classification system for this *parasport* prescribed by the *IWBF* (International Wheelchair Basketball Federation Executive Counsil, 2024), which categorizes players into eight classes (1.0, 1.5, 2.0, 2.5, 3.0, 3.5, 4.0, and 4.5). At the beginning of the study, the total sample included sixty (N = 60) wheelchair basketball players who, according to the competition categorization established by the *IWBF*, competed in Division B or C and had at least three years of competitive experience in this parasport. The age of the participants ranged from 16 to 54 years (median value= 37). Due to the expected attrition of the sample during the research process, testing of basic motor abilities was ultimately conducted on a total of fifty-two (N = 52) wheelchair basketball players, and only their results were included in the subsequent analyses. Among all participants, paraplegia was the most prevalent type of physical disability, observed in 22 wheelchair basketball players, with a higher representation among athletes competing in Division B (14 wheelchair basketball players). The least common type of disability was arthrogryposis, present in a single wheelchair basketball player participating in Division B (**Table 1**).

**Table 1:** Health status of wheelchair basketball players in relation to the type of physical disability.

Disability	Division B+C	Division B	Division C
Amputation	15	6	9
Paraplegia	22	14	8
Cerebral palsy	3	1	2
Paraparesis	4	1	3
Spina bifida	4	4	/
Arthrogryposis	1	1	/
Lower leg injury	3	1	2

Before data collection, an *a priori* sample size estimation was conducted using G*Power 3 (Faul et al., 2007). For the primary between-group comparisons between Division B and Division C players, both in the overall sample and within the lower (1.0–2.5) and higher (3.0–4.5) classification-score groups (one-way ANOVA, two groups), a significance level of α = 0.05 (95% confidence) and a desired statistical power of 0.80 (80% probability of detecting a true effect) were applied, assuming a medium effect size (Cohen’s f = 0.25, equivalent to d ≈ 0.50). A medium effect size was selected because previous wheelchair basketball studies reported group differences in motor and performance-related variables, but with variation in samples and testing protocols (de Witte et al., 2016; Granados et al., 2015; Yüksel and Sevindi, 2018). Based on these parameters, a total of 50 participants were required in the study. Based on this calculation, the study was sufficiently powered for the planned comparisons between Division B and Division C players.

Basic morphological characteristics of the sample were presented through measurements of seated height and body mass, obtained using a digital scale Seca 959 (Seca Instruments Ltd., Hamburg, Germany). Initially, participants were divided into two groups according to competitive rank (Division B and Division C). For more detailed analysis, participants were further stratified within each division according to classification score and functional trunk stability: one group comprised players with low classification scores (1.0–2.5) and the other comprised players with high classification scores (3.0–4.5). This grouping was used because lower classification scores (1.0–2.5) indicate lower trunk stability, while higher scores (3.0–4.5) indicate greater functional trunk stability in wheelchair basketball players.

- The first subsample (BDVO) comprised 13 wheelchair basketball players with high scores (3.0–4.5) who represent their national teams in Division B.
- The second subsample (BDNO) comprised 15 wheelchair basketball players with low scores (1.0–2.5) who represent their national teams in Division B.
- The third subsample (CDVO) comprised 13 wheelchair basketball players with high scores (3.0–4.5) who represent their national teams in Division C.
- The fourth subsample (CDNO) comprised 11 wheelchair basketball players with low scores (1.0–2.5) who represent their national teams in Division C.

In the European wheelchair basketball system, Divisions A, B, and C all represent elite international national-team competition, although Divisions B and C are lower competitive tiers compared with Division A (International Wheelchair Basketball Federation Executive Council, 2024; Yüksel and Sevindi, 2018). After a review and consultation with experts in parasport, participants provided written informed consent to participate in the study. All procedures were approved by the Ethics Committee of the Faculty of Sport and Physical Education, University of Niš (No. 04-111/2), and the study was conducted in accordance with the Declaration of Helsinki.

### 2.2 Procedures

#### 2.2.1 Study design

Testing of all participants in this study was conducted under conditions appropriate for wheelchair basketball competition, i.e., in sports facilities where the national teams, of which the study participants were members, carried out preparatory activities immediately prior to the European Championship Men Division B and C (ECMBC 2022). Participant testing was performed in the morning hours (09:00–12:00), and the sequence and timing of measurements were identical for all participants. The sports halls used for testing were well illuminated, adequately heated, clean, and spacious. Prior to testing, participants completed a 10-minute warm-up as part of their regular training routine. All measurements were performed by the same researches in the same order, and with the same instruments. On the day before testing, participants were informed about the characteristics of the tests and the testing schedule. A standardized familiarization procedure was conducted as part of the study, during which the researchers explained and demonstrated each test before formal data collection.

### 2.3 Measuring instruments

#### 2.3.1 Wheelchair basketball field tests

For all tests involving a start line, the front wheels of the wheelchair were positioned immediately behind the line, as close as possible without crossing it, unless a specific distance was required by the test protocol. For the assessment of basic motor abilities, wheelchair basketball field tests that have been used in previous studies by experts in the field (Marszalek et al., 2019b; Gerodimos, 2012; Yanci et al., 2015; Vanlandewijck et al., 1999; Marszalek et al., 2019b) were employed. The tested abilities were speed, strength, and agility of wheelchair basketball players. For assessment of wheelchair speed the following variables and tests were used: wheelchair propulsion over 5 m (5 m sprint test – ST5m), 10 m (10 m sprint test – ST10m), and 20 m (20 m sprint test – ST20m).

Sprint test at 5 m, 10 m and 20 m (ST5m, ST10m, ST20m) – Speed testing was performed with the participants seated in the wheelchair with the feet placed in the footrests. The large wheels of the wheelchair were positioned immediately behind the start line. The participants were instructed to propel the wheelchair by hand as fast as possible over a distance of 20 m, while sensors of a wireless photocell timing system (Photocells Microgate) recorded split times and results at 5 m, 10 m, and 20 m. Results were recorded in seconds (s), and the best time from two attempts was taken as the final score for each variable, with at least 120 s of rest between attempts The validity and reliability (ICC = 0.94 for ST5m, ICC = 0.96 for ST10m, and ICC = 0.97 for ST20m; where validity was examined against Wingate Anaerobic Test parameters in players classified as 1.0–2.5 and 3.0–4.5) of this test were confirmed by Marszalek et al. (2019b). Wingate-based validity was interpreted as validity evidence for anaerobic performance, not as direct validation of isolated speed, strength, or agility abilities.

For the assessment of upper-limb strength, the following variables and tests were used: basketball chest pass (Basketball Chest Pass Test – BCPT), medicine ball chest pass (Medicine Ball Chest Pass Test – MBTT), and maximal grip strength of the dominant hand (Handgrip Test – HT).

Basketball Chest Pass Test and Medicine Ball Chest Pass Test (BCPT and MBTT) – Both tests were performed with the participants were seated in the wheelchair at the center of the baseline, with the feet placed in the footrests and positioned so that the front wheels were behind the line. The participants were required to perform a two-handed chest pass from a static position, projecting either a basketball (BCPT) or a 3 kg medicine ball (MBTT) as far as possible, while one of the researchers stabilized the wheelchair from behind. The distance was measured from the baseline to the point at which the ball first contacted the floor. Four experienced assessors were present during the measurement procedure, and the final landing point and recorded distance were determined by consensus after each attempt. The result was expressed in meters (m) and represented the best value recorded from three trials for each test, with at least 120 s of rest between trials. The validity and reliability of these tests were confirmed in previous wheelchair basketball research in elite male players, with test–retest reliability reported for the BCPT (ICC = 0.94) and the MBTT (ICC = 0.97); validity was examined through associations with Wingate Anaerobic Test parameters (Marszalek et al., 2019b).

Handgrip Test of the Dominant Hand (HT) – The test was performed in a seated position in the wheelchair. The participants executed a maximal handgrip contraction with the fully extended dominant arm, which was measured using a handheld dynamometer (Jamar, JAP). The measurement was repeated three times with a rest interval of at least 60 s between trials, and the highest value was used to determine maximal strength. Visual feedback of the recorded force was provided. The result used for analysis was the maximal isometric contraction force sustained over five seconds, expressed in newtons (N). The reliability of this test was confirmed in the study by Gerodimos (2012), who reported high test–retest reliability for the preferred hand (ICC = 0.94–0.98) in basketball players. Validity data for the HT were not available in the cited study, and this should be considered when interpreting this measure.

For the assessment of agility, the best achieved values from three different agility tests were used. These tests differed in movement patterns, complexity, and duration and included the Agility T-test (Agility T-test – ATT), the Figure Eight Test (Figure Eight Test – FET), and the Drill Agility Test (DAT).

**Figure 1:**
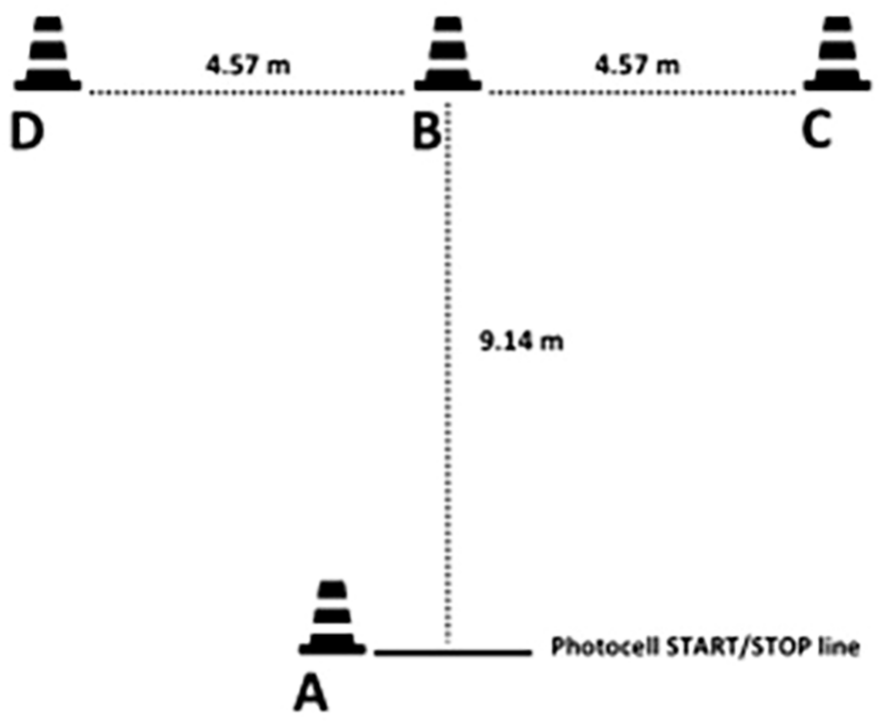
Schematic representation of the T-test agility setup on a basketball court.

Agility T-test (ATT) – The test was performed with participants positioned in their wheelchairs with the wheels placed 0.5 m behind the line where the cone at point A was located, and the test was executed by wheelchair propulsion forward in the manner illustrated in **Figure 1**. The distance between points A and B was 9.14 m, and during movement each participant was required to touch the top of the cone at point B, followed by the cone at point C, which was located at a distance of 4.57 m (**Figure 1**). The distance between the cones at points C and D was 9.14 m, and participants were required to touch the top of the cone at point D, then repeat the same procedure when returning to point B, and finally pass through the start/finish line at maximum speed at point A, where a wireless photocell system for electronic time measurement (Photocells Microgate) was positioned. All participants performed the test three times, with at least 180 s of rest between trials. The total distance covered was 36.56 m, and the cone height was 0.3 m. Timing started and ended when the participants crossed the line where the electronic timing photocells were positioned. The reliability of this test was confirmed in the study by Yanci et al. (2015) in wheelchair basketball players from a national league (ICC = 0.74).

**Figure 2:**
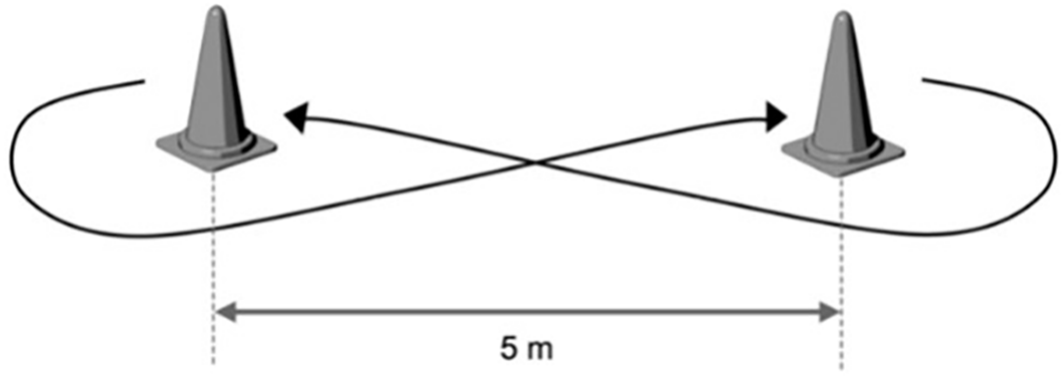
Schematic representation of the figure eight test setup on a basketball court.

Figure Eight Test (FET) – The test was performed with the participants assuming the starting position as illustrated in **Figure 2**. Following the given signal, the participants propelled the wheelchair around two cones, tracing a “figure-eight” movement pattern on the court for a duration of one minute. Participants were instructed to pass as close as possible to each cone during every turn in order to standardize the trajectory and minimize variations in turning strategy. The cones were placed 5 m apart, symmetrically in relation to the center line of the basketball court. The participant’s result was expressed as the total number of completed 5 m distances. If the final signal, given by the assessors, occurred while the participants were beyond the center line of the basketball court when returning toward the starting line, this distance was considered valid and included in the final result. The Figure Eight Test has been used as part of a wheelchair basketball field-test battery in previous research conducted with male wheelchair basketball players, where field tests were evaluated in relation to aerobic, anaerobic, and wheelchair basketball skill performance (Vanlandewijck et al., 1999). However, specific reliability and validity data for this test were not reported in that study, which should be considered when interpreting the FET results.

**Figure 3:**
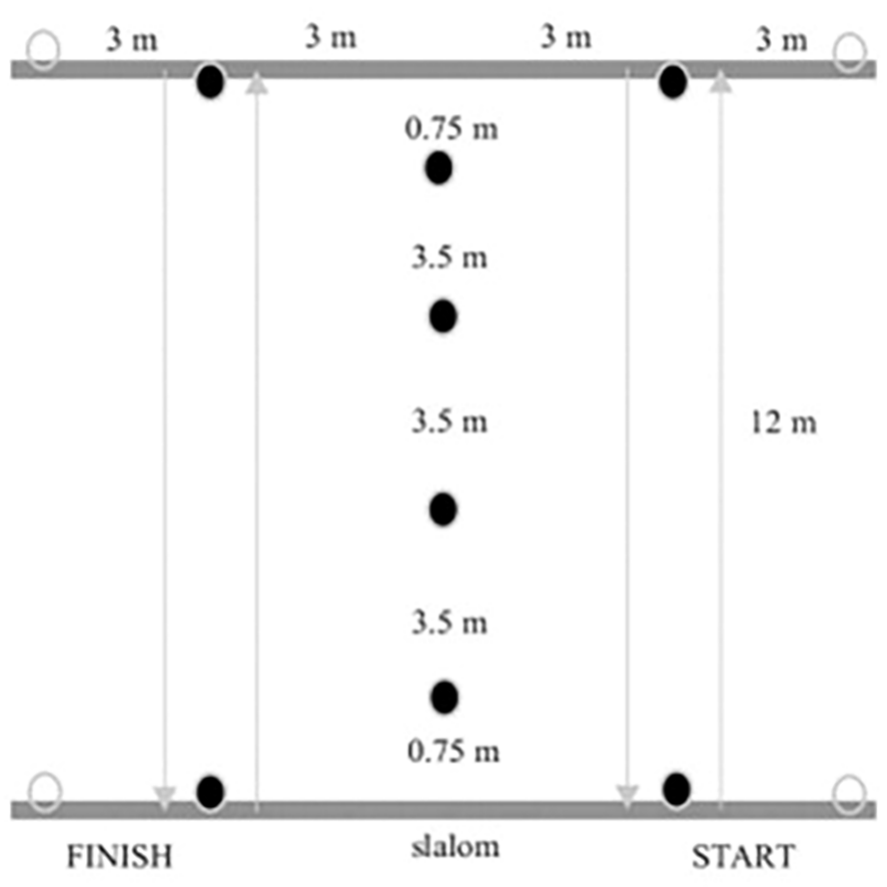
Schematic representation of the drill agility test setup on a basketball court.

Drill Agility Test (DAT) – The test is performed with participants positioned immediately behind the starting line, as close as possible without crossing it, with their hands on the wheels of the chair. They propel themselves as quickly as possible to execute a turn around a cone placed 12 meters directly in front of them. Following the turn, participants return and complete a slalom course weaving between four cones in both directions. Subsequently, they navigate around a cone positioned on the opposite side, also at a distance of 12 meters, and the test concludes as they cross the finish line opposite the starting position (**Figure 3**). The final score is determined by the better completion time of two attempts, with at least 120 s of rest between attempts. Completion time was measured using Witty photocells, with timing starting when the participants crossed the start line and stopping when the participants crossed the finish line. The validity and reliability of this test have been examined in previous wheelchair basketball research in elite male players, with very high test–retest reliability reported for the DAT (ICC = 0.99), although a significant test–retest difference was also observed (p = 0.015), indicating that this result should be interpreted cautiously (Marszalek et al., 2019b).

#### 2.3.2 Statistical analysis

All obtained data were presented using descriptive statistics parameters (mean, standard deviation – SD, minimum – min, and maximum – max). The Kolmogorov–Smirnov test was applied to assess the normality of the data distribution. Group differences were first examined at the multivariate level using multivariate analysis of variance (MANOVA), in order to test whether the groups differed across the combined set of motor ability variables. When a significant multivariate effect was observed, one-way analysis of variance (ANOVA) was then used to identify differences in individual variables. A significance level of *p* < 0.05 was applied to determine statistical significance between groups. Data analysis was performed using the SPSS statistical software package (IBM Corp., 2010; IBM SPSS Statistics for Windows, Version 19.0, Armonk, NY: IBM Corp.).

## 3 Results

**Table 2** presents the classification scores of the participants in the study, specifically wheelchair basketball players from Divisions B and C, both before and after subdivision into subgroups based on trunk functional stability.

**Table 2:** Classification scores of wheelchair basketball players in B and C divisions.

Classification score	Division B+C (n=52)	Division B (n=28)		Division C (n=24)	
		High scores (n=13)	Low scores (n=15)	High scores (n=13)	Low scores (n=11)
1.0	9	/	4	/	5
1.5	7	/	4	/	3
2.0	6	/	5	/	1
2.5	4	/	2	/	2
3.0	5	3	/	2	/
3.5	4	1	/	3	/
4.0	8	6	/	2	/
4.5	9	3	/	6	/

**Table 3** presents the primary descriptive parameters (mean, standard deviation – SD, minimum – Min, and maximum – Max) of the morphological characteristics and performance outcomes in basic motor ability tests of the participants (Divisions B + C, *n* = 52), as well as the results of participants divided according to the divisions in which they compete (Division B, *n* = 28; Division C, *n* = 24).

**Table 3:** Descriptive parameters of participants.

	Division B+C (n=52)			Division B (n=28)			Division C (n=24)		
Variables	Mean ± SD	Min	Max	Mean ± SD	Min	Max	Mean ± SD	Min	Max
Age (years)	36.56 ± 9.27	16.00	54.00	37.46 ± 10.14	16.00	52.00	35.50 ± 8.22	19.00	54.00
SH (cm)	94.44 ± 6.55	80.60	112.00	95.11 ± 7.49	80.60	112.00	93.67 ± 5.29	84.40	103.90
BM (kg)	81.17 ± 15.32	50.00	118.00	82.10 ± 15.15	50.00	110.70	80.09 ± 15.77	56.70	118.00
ST5m (s)	1.93 ± 0.19	1.48	2.36	1.86 ± 0.18	1.48	2.20	2.02 ± 0.18	1.65	2.36
ST10m (s)	3.27 ± 0.29	2.30	3.71	3.16 ± 0.29	2.30	3.62	3.39 ± 0.24	2.90	3.71
ST20m (s)	5.63 ± 0.42	4.82	6.51	5.46 ± 0.38	4.82	6.14	5.82 ± 0.38	5.13	6.51
BCPT (m)	10.56 ± 1.93	7.00	16.90	10.75 ± 1.62	7.50	13.90	10.34 ± 2.26	7.00	16.90
MBTT (m)	6.13 ± 1.19	3.95	9.75	6.21 ± 1.09	4.00	8.80	6.03 ± 1.31	3.95	9.75
HT (N)	513.71 ± 109.75	272.00	845.00	511.36 ± 87.35	351.00	760.00	516.46 ± 133.18	272.00	845.00
АТТ (s)	14.97 ± 1.27	13.14	18.33	14.50 ± 1.00	13.14	16.15	15.51 ± 1.35	13.45	18.33
DAT (s)	29.63 ± 2.37	25.30	37.41	28.46 ± 1.48	25.30	30.85	30.98 ± 2.51	26.56	37.41
FЕТ	17.96 ± 1.56	14.00	20.00	18.71 ± 1.12	16.00	20.00	17.08 ± 1.56	14.00	20.00

Mean ± SD, arithmetic mean ± standard deviation; Min, minimum result; Max, maximum result; SH, sitting height; BM, body mass; ST5m, 5m sprint test; ST10m, 10m sprint test; ST20m, 20m sprint test; BCPT, Basketball chest pass test; MBTT, Medicine ball chest pass test; HT, Handgrip test of the dominant hand; АТТ, Agility Т-test; DAT,Drill agility test; FЕТ, Figure eight test.

Using the Kolmogorov–Smirnov test, an analysis of the descriptive parameters of basic motor abilities in wheelchair basketball players was conducted, which revealed no significant deviations and confirmed the assumption of normal data distribution (*p>0.05*). The results of the analysis indicate a statistically significant intergroup difference at the multivariate level (*p* = 0.01) between players in Divisions B and C in the parameters of basic motor abilities. At the univariate level, significant intergroup differences (*p* < 0.05) were observed between players from Divisions B and C in wheelchair sprint performance over 5 m, 10 m, and 20 m, as well as in the ATT, DAT, and the “FAT” with Division B players achieving faster sprint and change-of-direction times, and a higher number of completed distances in FET. No statistically significant differences were found between groups in upper-body strength assessments (**Table 4**).

**Table 4:** Differences in basic motor skills between wheelchair basketball players of division B and C at the univariate level (ANOVA) and multivariate level (MANOVA).

Variables	Division B	Division C	Mean Square	F	p
ST5m (s)	1.86 ± 0.18	2.02 ± 0.18	0.35	10.95	**0.00**
ST10m (s)	3.16 ± 0.29	3.39 ± 0.24	0.72	10.27	**0.00**
ST20m (s)	5.46 ± 0.38	5.82 ± 0.38	1.70	11.88	**0.00**
BCPT (m)	10.75 ± 1.62	10.34 ± 2.26	2.18	0.58	0.45
MBTT (m)	6.21 ± 1.09	6.03 ± 1.31	0.42	0.29	0.59
HT (N)	511.36 ± 87.35	516.46 ± 133.18	336.29	0.03	0.87
АТТ (s)	14.50 ± 1.00	15.51 ± 1.35	13.32	9.62	**0.00**
DAT (s)	28.46 ± 1.48	30.98 ± 2.51	82.10	20.10	**0.00**
FЕТ	18.71 ± 1.12	17.08 ± 1.56	34.37	19.19	**0.00**
МАNOVA	**Wilks’ Lambda=0.61, F(9, 42)=2.99, p=0.01**				

SH, sitting height; BM, body mass; ST5m, 5m sprint test; ST10m, 10m sprint test; ST20m, 20m sprint test; BCPT, Basketball chest pass test; MBTT, Medicine ball chest pass test; HT, Handgrip test of the dominant hand; АТТ, Agility Т-test; DAT,Drill agility test; FЕТ, Figure eight test; ANOVA, difference value at the univariate level; MANOVA, difference value in basic motor skills at the multivariate level.

Bold values indicate statistically significant differences (p < 0.05).

**Table 5** presents the differences in basic motor abilities between wheelchair basketball players with lower classification scores in Divisions B and C, analyzed at both the univariate and multivariate levels. At the multivariate level, a statistically significant intergroup difference was observed in the examined basic motor ability parameters (*p* = 0.01). At the univariate level, statistically significant differences (*p* < 0.05) were identified between these players in wheelchair sprint performance over 5 m and 20 m, as well as in the ATT, DAT, and the FAT, with lower-classification Division B players achieving faster ST5m, ST20m, ATT, and DAT times, and higher FET scores. No statistically significant differences were found in wheelchair sprint performance over >10 m, upper-body strength assessments; upper-body strength assessments.

**Table 5:** Differences in basic motor skills between wheelchair basketball players with lower classification scores in division B and C at the univariate level (ANOVA) and multivariate level (MANOVA).

Variables	Division B	Division C	Mean Square	F	P
ST5m (s)	1.94 ± 0.18	2.07 ± 0.09	0.10	4.61	**0.04**
ST10m (s)	3.27 ± 0.34	3.46 ± 0.14	0.25	3.30	0.08
ST20m (s)	5.62 ± 0.40	5.95 ± 0.25	0.71	6.03	**0.02**
BCPT (m)	9.92 ± 1.39	9.10 ± 1.14	4.22	2.51	0.13
MBTT (m)	5.69 ± 0.99	5.41 ± 0.84	0.49	0.56	0.46
HT (N)	484.27 ± 81.04	544.45 ± 138.45	22989.45	1.94	0.18
АТТ (s)	14.67 ± 1.00	15.63 ± 1.10	5.84	5.35	**0.03**
DAT (s)	28.86 ± 1.75	32.25 ± 2.06	73.00	20.53	**0.00**
FЕТ	18.53 ± 0.91	16.54 ± 0.93	25.08	29.42	**0.00**
МАNOVA	**Wilks’ Lambda=0.32, F(9, 16)=3.77, p=0.01**				

SH, sitting height; BM, body mass; ST5m, 5m sprint test; ST10m, 10m sprint test; ST20m, 20m sprint test; BCPT, Basketball chest pass test; MBTT, Medicine ball chest pass test; HT, Handgrip test of the dominant hand; АТТ, Agility Т-test; DAT,Drill agility test; FЕТ, Figure eight test; ANOVA, difference value at the univariate level; MANOVA, difference value in basic motor skills at the multivariate level.

Bold values indicate statistically significant differences (p < 0.05).

Differences in basic motor abilities between wheelchair basketball players with higher classification scores in Divisions B and C at both the univariate and multivariate levels are presented in **Table 6**. No statistically significant differences were observed between groups at the multivariate level in the examined basic motor ability parameters (*p* = 0.24). At the univariate level, statistically significant differences (*p* < 0.05) were identified between groups in wheelchair sprint performance over 5 m, 10 m, and 20 m, as well as in agility tests, including the ATT, DAT, and the “FAT, with higher-classification Division B players achieving faster sprint and change-of-direction times, and higher FET scores. No statistically significant intergroup differences were found in upper-body strength assessments.

**Table 6:** Differences in basic motor skills between wheelchair basketball players with high classification scores in division B and C at the univariate level (ANOVA) and multivariate level (MANOVA).

Variables	Division B	Division C	Mean Square	F	P
ST5m (s)	1.76 ± 0.12	1.98 ± 0.22	0.32	9.80	**0.01**
ST10m (s)	3.03 ± 0.14	3.33 ± 0.29	0.60	11.6	**0.00**
ST20m (s)	5.28 ± 0.26	5.71 ± 0.44	1.22	9.192	**0.01**
BCPT (m)	11.71 ± 1.34	11.38 ± 2.47	0.68	0.17	0.68
MBTT (m)	6.81 ± 0.89	6.55 ± 1.43	0.43	0.30	0.59
HT (N)	542.61 ± 86.77	492.77 ± 129.22	16150.15	1.33	0.26
АТТ (s)	14.30 ± 1.00	15.41 ± 1.58	8.09	4.64	**0.04**
DAT (s)	28.01 ± 0.99	29.92 ± 2.41	23.56	6.93	**0.02**
FЕТ	18.92 ± 1.32	17.54 ± 1.85	12.46	4.81	**0.04**
МАNOVA	Wilks’ Lambda=0.55, F(9, 16)=1.47, p=0.24				

SH, sitting height; BM, body mass; ST5m, 5m sprint test; ST10m, 10m sprint test; ST20m, 20m sprint test; BCPT, Basketball chest pass test; MBTT, Medicine ball chest pass test; HT, Handgrip test of the dominant hand; АТТ, Agility Т-test; DAT,Drill agility test; FЕТ, Figure eight test; ANOVA, difference value at the univariate level; MANOVA, difference value in basic motor skills at the multivariate level.

Bold values indicate statistically significant differences (p < 0.05).

## 4 Discussion

The present study identified differences in basic motor abilities between wheelchair basketball players from Divisions B and C, with the clearest differences observed in speed and change-of-direction speed, while strength-related tests did not differ significantly between groups. These differences were more evident among players with lower classification scores, whereas players with higher classification scores did not differ significantly at the multivariate level. Therefore, the main finding of this study is that competitive level appears to be more clearly reflected in wheelchair sprint and change-of-direction performance than in the applied strength tests. The present findings should be interpreted within current wheelchair basketball field-test research, where sprint, change-of-direction, passing, and wheelchair-mobility tests are commonly used to evaluate performance in relation to competitive level and functional classification (Ferreira da Silva et al., 2022; Petrigna et al., 2022; Hernández-Beltrán et al., 2024b).

Overall, the comparison of Division B and Division C players showed a significant multivariate difference in the combined motor-performance profile, indicating that competitive level was associated with the overall pattern of tested abilities. This finding is consistent with previous research showing that higher competitive level is related to better wheelchair basketball field-test performance (Granados et al., 2015), and it is also supported by recent match-analysis evidence showing that higher-performing wheelchair basketball teams differ in relevant performance indicators and class-composition patterns at the international level (Lee and Kim, 2025). However, the differences observed in the present study were not equally distributed across all motor domains. They were most evident in sprint performance, where Division B players achieved faster results in ST5m, ST10m, and ST20m. These sprint tests have been commonly used in previous wheelchair basketball research (Gil et al., 2015; Yanci et al., 2015; Marszałek et al., 2019a), and their relevance is supported by the intermittent nature of the sport, which includes repeated accelerations, stops, and short-distance wheelchair propulsion actions (Vanlandewijck et al., 1999; Wang et al., 2005; Marszałek et al., 2019a). Similar differences were found in change-of-direction speed, where Division B players performed better in ATT, DAT, and FET. This is in line with the view that the ability to manoeuvre and rapidly change direction while propelling the wheelchair is an important component of wheelchair basketball performance (Ozmen et al., 2014), and with previous work in which these types of field tests were used to assess wheelchair mobility and performance (Vanlandewijck et al., 1999; Gil et al., 2015; Granados et al., 2015; Yanci et al., 2015; Marszałek et al., 2019a; Tachibana et al., 2019). Recent systematic and mini-review evidence also confirms that sprint, change-of-direction, passing, and wheelchair-mobility tests remain among the most frequently used field assessments in wheelchair basketball (Ferreira da Silva et al., 2022; Petrigna et al., 2022), while recent wheelchair basketball studies further show that performance interpretation should consider functional classification and the specific competitive context of the sport (Hernández-Beltrán et al., 2024a, b; Lee and Kim, 2025), which supports the relevance of the test battery used in the present study for examining differences between competitive levels and functional-classification groups. In contrast, BCPT, MBTT, and HT did not differ significantly between Divisions B and C. Although Division B players had descriptively higher throwing results in BCPT and MBTT, Division C players showed higher values in HT, which may be partly explained by the presence of athletes who also participate in other para-sports where strength is a dominant ability. Therefore, the absence of significant differences in the applied strength tests should not be interpreted as evidence that strength is unimportant, but rather that these tests may not have captured sport-specific strength demands as clearly as the sprint and change-of-direction tests, especially since upper-limb strength has been linked to repeated change-of-direction ability in international-level wheelchair basketball players (Iturricastillo et al., 2022).

The specificity of the present study is reflected, among other aspects, in the subdivision of wheelchair basketball players into two subgroups within a single competitive division based on classification scores (low classification scores: 1.0–2.5; high classification scores: 3.0–4.5), according to trunk functional stability, as performed in previous studies (Molik et al., 2010b; Zacharakis et al., 2012; Yanci et al., 2015; de Witte et al., 2016; Marszałek et al., 2019a, b). This approach is consistent with recent research in which low-point and high-point players are analyzed separately because classification score reflects functional ability, trunk control, and the player’s volume of action (Hernández-Beltrán et al., 2024b; Farì et al., 2025). Differences in basic motor abilities between wheelchair basketball players with lower classification scores in Divisions B and C at the multivariate level are presented in **Table 5**. Multivariate analysis of variance revealed a statistically significant difference (Wilks’ Lambda = 0.32, F(9, 16) = 3.77, *p* = 0.01) between players with lower classification scores in Division B (*n* = 15) and those in Division C (*n* = 11) regarding the combination of variables describing the basic motor ability domain. This difference was mainly in favor of lower-classification Division B players, who showed better sprint and change-of-direction performance than lower-classification Division C players. The clearer difference among low-classification players may be related to reduced trunk stability, which can limit balance, force transfer, and wheelchair control. Therefore, better propulsion and turning efficiency in Division B players may produce larger differences in this subgroup (Molik et al., 2010; Marszałek et al., 2019b; García-Fresneda et al., 2022). Previous studies have tested motor abilities of wheelchair basketball players at different competitive levels (Ergun et al., 2008; Erdemİr et al., 2009; De Groot et al., 2012; Granados et al., 2015; Yüksel and Sevindi, 2018), but the samples at each competitive level included players across all classification scores, without subdivision into low and high scores based on trunk functional stability. Thus, beyond the differences observed in the criteria and subgroup division applied in this study, compared to previous research assessing motor abilities at different competitive levels, the present study also differs in the quantity of applied tests. In this context, multivariate-level comparisons cannot be directly made with previous studies, and the analysis of the tested motor domains must therefore be conducted at the univariate level. However, findings should also be interpreted with caution because recent research has shown that functional classification does not always correlate clearly with every field-based performance outcome, suggesting that classification score is important but not the only factor explaining performance variation (Ceruso et al., 2022).

The review of individual results in the univariate analysis of basic motor ability tests (**Table 5**) revealed statistically significant differences between wheelchair basketball players with lower classification scores in Divisions B and C in wheelchair sprint tests over 5 m (ST5m, *p* = 0.04) and 20 m (ST20m, *p* = 0.02). Overall, sprint-test outcomes in this subgroup were generally better in Division B players, although not all individual sprint distances reached statistical significance. Compared with previous studies, Division B players with classification scores between 1.0 and 2.5 demonstrated superior 20 m non-ball sprint performance compared with Greek national team players (Zacharakis et al., 2012). In addition, Division B players also performed better in the 5 m and 10 m non-ball sprints, while achieving slightly lower average results in the 20 m sprint (by 0.05 s) relative to players with corresponding classification scores from the national teams of Poland, Latvia, Lithuania, and France (Marszałek et al., 2019b). Recent research on elite wheelchair basketball players has also shown that initial maximum push-rim propulsion is associated with sprint performance and differs according to functional classification, which provides a possible biomechanical explanation for the importance of short-distance acceleration in this sport (García-Fresneda et al., 2022). Similar trends, without statistically significant differences, were observed in strength tests across the total sample of Divisions B and C after subdivision into subgroups based on trunk functional stability. The Division B subgroup with lower classification scores achieved notably higher results in MBTT tests compared with the Division C subgroup (BCPT, Mean ± SD: 9.92 ± 1.39 m > 9.10 ± 1.14 m; MBTT, Mean ± SD: 5.69 ± 0.99 m > 5.41 ± 0.84 m). No statistically significant intergroup difference was observed in maximal handgrip strength (*p* = 0.18), although individual Division C players who participate in other para-sports contributed to higher group values in this test (HT, Mean ± SD: 544.45 ± 138.45 N > 484.27 ± 81.04 N). Although the global MANOVA did not reach significance, individual ANOVAs indicated statistically significant intergroup differences between low-classification players in Divisions B and C in all agility tests (ATT, p = 0.03; DAT, p < 0.001; FET, p < 0.001) with Division B players achieving faster ATT and DAT times and higher FET scores. consistent with the findings of Granados et al. (2015), who reported greater agility in players competing in higher divisions. As noted by Goosey-Tolfrey (2010), agility, the ability to manoeuvre the wheelchair efficiently, is fundamental for wheelchair basketball performance, which explains the advantage observed in these agility test results among players competing in the higher-quality division. This interpretation is further supported by recent work showing that wheelchair basketball performance depends not only on isolated physical capacity, but also on the player’s ability to repeatedly respond to sport-specific movement demands during training and competition (Romarate et al., 2023; Hernández-Beltrán et al., 2024a).

Regarding high classification players, at the multivariate level, no statistically significant intergroup difference was observed (Wilks’ Lambda = 0.55, F(9, 16) = 1.47, *p* = 0.24). Therefore, the univariate findings in this subgroup should be interpreted cautiously and treated as descriptive indicators rather than as strong evidence of a global difference between high-classification players from the two divisions. The weaker multivariate difference among high-classification players may be partly explained by their greater trunk stability and mobility. In this group, performance may depend more on tactical role, training history, wheelchair setup, and sport-specific experience than on classification alone (de Witte et al., 2016; Hernández-Beltrán et al., 2024b; Lee and Kim, 2025). At the univariate level, however, Division B players with high scores outperformed their Division C counterparts in the majority of variables (six of nine). Statistically significant intergroup differences were found in all applied wheelchair sprint tests (ST5m: *p* = 0.01; ST10m: *p* < 0.001; ST20m: *p* = 0.01) and in agility tests (T-test, ATT: *p* = 0.04; Drill Agility Test, DAT: *p* = 0.02; figure-eight, FET: *p* = 0.04), with higher-classification Division B players achieving faster sprint and change-of-direction times and higher FET scores than higher-classification Division C players. The mean DAT performance for high-score Division B players (Mean ± SD: 28.01 ± 0.99 s) exceeded the values reported by Marszałek et al. (2019b). Strength tests showed no significant intergroup differences (BCPT: *p* = 0.68; MBTT: *p* = 0.59; HT: *p* = 0.26), although Division B players recorded descriptively higher means in all strength measures (BCPT, Mean ± SD: 11.71 ± 1.34 m > 11.38 ± 2.47 m; MBTT, Mean ± SD: 6.81 ± 0.89 m > 6.55 ± 1.43 m; HT, Mean ± SD: 542.61 ± 86.77 N > 492.77 ± 129.22 N). Notably, sprint and agility outcomes for high-classification Division B players were, on average, superior to corresponding values reported in Marszałek et al. (2019b) and Zacharakis et al. (2012), whereas throwing results for high-score B players were, on average, lower than those reported by Marszałek et al. (2019b). These findings indicate that, despite the absence of a multivariate effect, meaningful univariate advantages in speed and agility characterize high-score players competing at the higher competitive level. Taken together, these findings suggest that high-classification players from Division B showed more favorable descriptive and univariate sprint and agility profiles, but the absence of a significant multivariate effect means that these results should not be overgeneralized. This pattern is compatible with recent evidence indicating that functional classification and performance are related in complex ways and should be interpreted together with training status, game role, tactical demands, and sport-specific wheelchair use (Ceruso et al., 2022; Hernández-Beltrán et al., 2024b; Lee and Kim, 2025).

Despite the contributions of this study to understanding the motor abilities of wheelchair basketball players across competitive levels and functional classifications, several limitations should be noted. The sample size, although representative of multiple national teams, was relatively small, particularly in subgroup analyses, which may limit the generalizability of the findings. Additionally, the cross-sectional design does not allow conclusions regarding causal relationships between classification, competitive level, and motor performance. Moreover, routine training processes, sleep and dietary habits, and stimulant use were not recorded, which could have influenced the study results and should be considered in interpreting the findings. Another limitation of the study is the lack of information regarding the type, brand, and configuration of wheelchairs used by the players, which may have influenced performance outcomes. Future research should employ longitudinal designs to examine training adaptations over time, incorporate larger and more diverse samples, and explore additional performance-related factors such as endurance, coordination, and cognitive-motor integration during gameplay. Additionally, future studies should also consider monitoring participants’ sleep, dietary habits, and stimulant use, as these factors may influence performance. Investigating the effects of targeted interventions on specific motor abilities may also provide practical insights for coaches and rehabilitation specialists aiming to optimize performance in wheelchair basketball.

## 5 Conclusion

The findings indicate that statistically significant multivariate differences (*p* < 0.05) exist in the tested domain of basic motor abilities (speed, strength, and agility) between the overall samples of Division B and C players within these divisions, as well as between players with lower classification scores (1.0–2.5). No significant multivariate differences were observed in players with higher classification scores (3.0–4.5) between the two divisions. At the univariate level, statistically significant differences (*p* < 0.05) were found in speed and agility tests in favor of Division B players compared to Division C players, both in the total sample and when results were analyzed according to trunk functional stability (high and low classification scores), whereas no significant differences were identified in the applied strength tests. Overall, these results suggest clear differences in most tested parameters of basic motor abilities, particularly in speed and agility, between wheelchair basketball players of different competitive levels. These findings suggest that speed and agility are the primary motor abilities distinguishing players across competitive levels, whereas strength differences are less noticeable. Practically, this indicates that coaches should emphasize targeted speed and agility training, particularly for lower levels of competition, while combining strength exercises with sport-specific wheelchair mobility drills to optimize performance.

## Data Availability

The raw data supporting the conclusions of this article will be made available by the authors, without undue reservation.
